# Exploring how non-clinical factors in childbirth care shape users’ experiences in public health facilities in rural Chiapas, Mexico: a qualitative study using the WHO health systems responsiveness framework

**DOI:** 10.1186/s12884-024-06357-7

**Published:** 2024-02-29

**Authors:** Zeus Aranda, Viviana Caamal, Mariana Montaño, Daniel Bernal, Sergio Meneses

**Affiliations:** 1 Partners In Health Mexico (Compañeros En Salud), Ángel Albino Corzo, Calle Primera Pte. Sur 25, Colonia Centro, 30370 Ángel Albino Corzo, Chiapas, México; 2 Escuela de Gobierno y Transformación Pública, Instituto Tecnológico y de Estudios Superiores de Monterrey, Ciudad de México, México; 3 Center for Health Systems Research, National Institute of Public Health, Cuernavaca, Morelos México

**Keywords:** Facility-based childbirth experience, Health systems responsiveness, Qualitative study, Mexico

## Abstract

**Introduction:**

Many Mexicans face barriers to receive delivery care from qualified professionals, especially indigenous and poor sectors of the population, which represent most of the population in the state of Chiapas. When access to institutional delivery care is an option, experiences with childbirth care are often poor. This underscores the need for evidence to improve the quality of services from the user’s perspective. The present study was conceived with the objective of understanding how non-clinical aspects of care shape women’s birthing experiences in public health institutions in Chiapas.

**Methods:**

We conducted an exploratory qualitative study. Data collection consisted in 20 semi-structured interviews to women who had delivered in a public health facility in Chiapas during the last six months prior to the interview. For the design of the interview guide we used the WHO health system responsiveness framework, which focus on the performance of the health system in terms of the extent to which it delivers services according to the “universally legitimate expectations of individuals” and focuses on the non-financial and non-clinical qualities of care. The resulting data were analyzed using thematic analysis methodology.

**Results:**

We identified a total of 16 themes from the data, framed in eight categories which followed the eight domains of the WHO health systems responsiveness framework: Choice of the provider and the facility, prompt attention, quality of basic amenities, access to social support, respectful treatment, privacy, involvement in decisions, and communication. We shed light on the barriers women face in receiving prompt care, aspects of health facilities that impact women’s comfort, the relevance of being provided with adequate food and drink during institutional delivery, how accompaniment contributes positively to the birthing experience, the aspects of childbirth that women find important to decide on, and how providers’ interpersonal behaviors affect the birthing experience.

**Conclusions:**

We have identified non-clinical aspects of childbirth care that are important to the user experience and that are not being satisfactorily addressed by public health institutions in Chiapas. This evidence constitutes a necessary first step towards the design of strategies to improve the responsiveness of the Chiapas health system in childbirth care.

**Supplementary Information:**

The online version contains supplementary material available at 10.1186/s12884-024-06357-7.

## Introduction

In Mexico, a large proportion of the population faces barriers to giving birth with a skilled health professional, especially women without health insurance, in conditions of poverty, and in the indigenous population [1]. Lack of access is heterogeneous across states, Chiapas being the state with the least access to skilled professionals for childbirth according to the 2012 National Health and Nutrition Survey, at a 60.5% compared to 94.4% nationally [2]. The state public health system is limited by the lack of facilities, healthcare personnel and medicines [3–5], partially as a result of the stagnation in health system financing from 2016 to 2022 [6]. In addition, the poor socioeconomic conditions of the majority of the inhabitants of Chiapas, with 75.5% of its population living in poverty [7], makes it difficult for the population to seek other healthcare options in the private sector when the public system is unavailable or unresponsive.

For women who can access childbirth care in health facilities in Mexico, many are victims of obstetric abuse and violence, as well as non-consensual care. According to the 2016 National Survey on the Dynamics of Household Relationship, 33.3% of Mexican women had experienced obstetric violence during their last childbirth [8]. In a study conducted in Chiapas surveying women who had given birth in a public facility in the capital, this figure rose to 49.2% [9]. This type of violence especially affects women living in vulnerable situations, such as indigenous populations [10], which represent 28.2% of the population of Chiapas [11]. In some cases, due to fear of mistreatment in healthcare facilities, women decide to give birth at home with the support of traditional birth attendants [12], sometimes hours away from the nearest health facility, with the risks that this may imply.

The lack of timely access to quality childbirth care is related to the high maternal and neonatal morbidity and mortality that Chiapas has experienced over the past decades [2]. In 2022, there were 35 maternal deaths per 100,000 live births in Chiapas according to official figures [13], although the actual figure is likely higher, as underreporting of maternal deaths to/by public institutions in the state has been previously identified [14, 15]. There is a need to improve access and quality of care to reduce maternal mortality and morbidity [16] and to guarantee the rights of women and newborns in the context of maternity care provided in healthcare facilities [17].

Quality of care should be assessed from the point of view of both the service provider and the user, so that strategies can be designed to improve it according to both sets of criteria [18]. To assess service quality from the user’s perspective, the World Health Organization (WHO) introduced its health system responsiveness framework in 2000 [19]. The concept of responsiveness measures the performance of the health system in terms of the extent to which it delivers services according to the “universally legitimate expectations of individuals” and focuses on the non-financial and non-clinical qualities of care [19, 20]. In the original WHO framework, the responsiveness concept included seven different domains: Choice of the provider and the facility, prompt attention, quality of basic amenities, access to social support, respectful treatment and communication, privacy, and involvement in decisions. A revised version in 2003 added an eighth domain: Communication [20, 21]. Although this framework has been used previously in studies of childbirth care in Ethiopia, Ghana, Thailand, and The Netherlands [22–26], its use has been limited, especially in qualitative studies (only one). To our knowledge, there is no previous study using this approach to assess childbirth care in the Latin American region.

Qualitative evidence on the experiences of facility-based childbirth in Chiapas is still scarce, with only a few studies that either focus on specific areas of care [27, 28] or on indigenous populations [12, 29, 30]. The present study was conceived with the objective of understanding users’ experiences, perceptions and preferences about non-clinical aspects of facility-based childbirth care in Chiapas, focusing in the eight domains of the revised WHO responsiveness framework from 2003 [20] and using a qualitative approach. This evidence will serve as input in the design of strategies to improve the Chiapas health system responsiveness in childbirth care.

## Methods

### Study setting

The study was led by researchers from the non-governmental organization (NGO) Compañeros En Salud (CES; as Partners In Health is known in Mexico), the Monterrey Institute of Technology, and the National Institute of Public Health of Mexico (INSP). CES has provided perinatal and maternal health services in the Fraylesca and Sierra regions of Chiapas since 2011. In 2016, the NGO introduced a respectful childbirth care model in a basic community hospital of the Ministry of Health (MOH) in Ángel Albino Corzo, in the Fraylesca region of Chiapas. In 2017, CES, with the support of the MOH and local authorities, built a birthing center adjacent to the basic community hospital, staffed by obstetric nurses with the respectful childbirth care model at the center [31]. In order to improve the quality of childbirth care delivered in the supported facilities and in the state, CES decided to join efforts with the Tec and the INSP to conduct the present study. Interviews were conducted in the localities of Ángel Albino Corzo, Francisco I. Madero, Honduras de la Sierra, Querétaro, Laguna del Cofre, and Reforma, all in the Fraylesca and Sierra regions of Chiapas, Mexico.

### Participants

Semi-structured interviews were conducted with twenty women who had given birth at the CES-supported birthing center and adjacent basic community hospital in Ángel Albino Corzo, Villaflores Bicentenario hospital, Motozintla hospital, and Siltepec hospital, all health facilities in the Fraylesca and Sierra regions of Chiapas, Mexico.

Participants were selected using convenience sampling. Due to logistical limitations, the individuals to be interviewed had to reside in one of the communities where the NGO leading the project, CES, has a presence. For this reason, it was decided to identify residents of these communities who had attended obstetric consultations in the final stage of pregnancy at the CES-supported birthing center in Ángel Albino Corzo in the last six months. A list was obtained with the telephone contacts of these users, which also included some basic demographic data, such as place of residence, age, and parity number. Women were contacted from the most recent to the oldest (to limit recall bias among participants) and were asked when and where they had given birth. Women who met the selection criteria were purposively sampled to obtain a sample with different places of residence, ages, number of births and health facilities where they gave birth. Inclusion criteria were: (1) over 18 years of age; (2) vaginal delivery of a live newborn in a public health facility in Chiapas in the six months prior to the interview; (3) and residence in one of the CES-supported communities in the Fraylesca and Sierra regions at the time of the interview (to facilitate travelling of the data collector using the NGO’s transportation means). Exclusion criteria were: (1) inability to give consent; and (2) speech or hearing impediments.

The original estimate of participants ranged from 15 to 30, based on the previous experiences of the research team. However, the final sample consisted of 20 individuals. Sampling ended when saturation of the information was reached, when co-authors found that adding additional participants led to diminishing returns and the data collected were sufficiently rich in diversity and depth considering the study population [32]. All women who were offered participation were read an informed consent letter, which included detailed information about the study, its objective, its potential benefits and risks, that the interviews will be recorded, how the research team would treat the data provided by the participants, and the voluntariness of participation with no negative consequences for those who chose not to participate. None of the women who were offered participation refused or dropped out of the study.

### Data collection

Interviews were conducted in July and August of 2022. Although some interviews were conducted at CES premises, most took place in the participant’s home. All interviews were conducted by the same data collector, a Mexican qualitative research expert with knowledge of the setting but no prior relationship with the participants. The interviews lasted between 60 and 90 min and were audio-recorded. In addition, the data collector took field notes to complement the recordings. The interviews were conducted in Spanish and transcribed with the support of a transcription software. Only selected excerpts were translated into English for publication with the collaboration of a native English speaker proficient in Spanish. The interviews followed semi-structured interview guides developed with the support of co-authors and health professionals outside the study. An initial version of the guide was piloted with one woman who met the selection criteria, which lead to the modification of the guide to make it more intelligible to the participants. The guide focused on the eight dimensions of the WHO responsiveness framework and included four sections: (1) Experiences at last childbirth; (2) preferences; (3) areas of improvement; and (4) preferred place of delivery. The interview guide can be found in Additional file 1.

### Data analysis

Transcripts were analyzed with the aid of Dedoose v. 9.0.62 software using the thematic analysis methodology as defined by Braun and Clarke [33]. After initial familiarization with the data, the first author and the data collector independently coded a subset of interviews. Discussion between the two researchers led to a consensus codebook, which was applied to the entire dataset. After coding the data, themes were identified following a deductive-inductive approach, as the themes emerging from the data were driven by the eight WHO responsiveness domains. The themes obtained were reviewed and refined through an iterative process until all co-authors were satisfied with the outcome. Participants did not provide feedback on the results. The authors adhered to the consolidated criteria for reporting qualitative research studies (COREQ) 32-item checklist, which can be found in Additional file 2.

## Results

Twenty women participated in the study. All the women were Mexican, non-indigenous and had at least completed primary education (Table 1). The median age of the participants was 29 years and the median number of births was 2.5. Most of the women had experienced childbirth before their last delivery (80%) and, of these, the majority only in health facilities (56.3%). Most of the women lived in the municipality of Ángel Albino Corzo (75%) and most were married or lived with their partner (85%). Most women gave birth in facilities coded as 1 (55%) and 2 (25%), with 1 being a basic community hospital and 2 a birthing center and the two being adjacent to each other.

**Table 1:** Sociodemographic characteristics of the participants interviewed

	*n* = 20	
	%	n
**Indigenous**		
No	100	20
**Nationality**		
Mexican	100	20
**Municipality of residence at time of interview**		
Ángel Albino Corzo (Fraylesca region)	75	15
Montecristo (Fraylesca region)	10	2
Honduras de la Sierra (Sierra Mariscal region)	10	2
La Concordia (Fraylesca region)	5	1
**Last school grade completed**		
Primary	35	7
Secondary	40	8
High school	25	5
**Marital status**		
Married	20	4
Living with a partner	65	13
Single	15	3
**Chilbirth experience prior to the last delivery**		
Yes	80	16
*Only in healthcare institutions*	56.3	9
*Only at home*	25	4
*In both*	6.3	1
No	20	4
**Facility of last childbirth**		
Facility 1 (Ángel Albino Corzo basic community hospital)*	55	11
Facility 2 (Ángel Albino Corzo birthing center)*	25	5
Facility 3 (Villaflores Bicentenario hospital)	10	2
Facility 4 (Siltepec hospital)	5	1
Facility 5 (Motozintla hospital)	5	1
	**Median**	**Interquartile range**
**Age at time of interview**	29	22.8–33
**Number of births**	2.5	1–4

*Adjacent facilities

The data are presented through the eight main categories, which correspond to the eight domains of the WHO health systems responsiveness framework [20, 34], and the 16 themes identified with illustrative excerpts from participants. Table 2 lists the categories and the themes associated with each category.

**Table 2:** Categories, corresponding to the eight domains of the WHO health system responsiveness framework, and themes identified after analysis

Category	Theme
**Choice of the provider and the facility**	Barriers to giving birth in a woman’s chosen facility
	Choice of healthcare provider based on user preferences
**Prompt attention**	Means of transportation and road conditions as determinants of prompt care
	Waiting time to initial evaluation upon arrival at the facility
	Referral to distant facilities poses a threat to the lives of mothers and babies
**Quality of basic amenities**	Main aspects that determine the comfort of pregnant women in the facility
	Access to food and beverages for users and their relatives
**Access to social support**	Accompaniment needs during childbirth and its positive aspects
**Respectful treatment**	Respectful care for women
	Respect and attention for pregnant women’s companions
**Privacy**	Exposure of women’s bodies to the gaze of strangers
**Involvement in decisions**	Informed consent before health providers perform clinical interventions
	Decision of other aspects that clinical interventions
**Communication**	Provision of information by healthcare providers
	Information requested by health providers at inappropriate times
	Communication between providers influences user experience

### Choice of the provider and the facility

#### Barriers to giving birth in a woman’s chosen facility

The reasons for choosing to give birth at a particular facility varied among women, although proximity to their home or that of their relatives was a major determining factor. Some women could choose between a birthing center and a basic community hospital next to each other. In this case, most women preferred to deliver at the birthing center, which follows respectful birthing practices. However, the hours of the birthing center, which was closed at night, or the presence of health risks to the mother or baby, sometimes resulted in women being referred to the adjacent hospital, which made them feel upset.

“*I was sad, I wanted to go there* [to the birthing center], *I already knew the place… whenever I arrived they always called my attention, they talked to me, everything, but they changed me to the hospital, I didn’t want to, but I didn’t tell them. I would have liked to have my baby at the birthing center.* […] *They* [the birthing center personnel] *had to close, they did not stay the whole shift, and with my previous baby they had worked all night, and that changed.*” (Participant 1, 30 years, facility 1).

In other cases, when complications arose during pregnancy, labor or childbirth, some women reported being referred to other facilities far from the one they had chosen due to a lack of specialists. This situation made the women feel concerned about who would look after their other children and about having to incur unforeseen expenses.

“*I just didn’t want that* [being transferred from the birthing center to facility 3], *I don’t want to move because of the children, my husband is here and he can go see them, they are close to here* […].” (Participant 2, 29 years, facility 1).

#### Choice of healthcare provider based on user preferences

In most cases, women were not offered the possibility of choosing the characteristics of the professional who would attend their delivery. Only two participants were allowed to choose between different midwives working at the facility and one woman was allowed to invite her traditional midwife from a neighboring community. Nevertheless, most participants felt it was important to choose the provider of their choice according to their preferences. For instance, some women expressed feeling more confident if they received care from someone they already knew from obstetric check-ups prior to delivery care or whom they had had the opportunity to meet during prenatal care visits. One user reflected this by stating:

*“Receiving care from complete strangers would have made me feel uncomfortable, because you don’t know how to start a conversation; on the other hand, she* [the nurse who attended her delivery] *had already taken care of me beforehand, she was the one who did the examination and was the one who admitted me to the hospital, the day before I had met her when I went to the health center to get my admission, she had already taken care of me and from there I already felt confident”.* (Participant 3, 33 years, facility 4)

Women appreciated being attended by the same professionals throughout the delivery process, in order to create a bond and have more confidence to express their concerns. Also, the interviewed users commonly expressed that they felt more comfortable being attended by a woman than by a man, and that this made them feel more confident and trusting and less shy. One women relayed:

“*Well, at the birthing center it’s calmer, you feel…. As I said, there are more nurses, you communicate more with women, with men it’s different because of the doctors, you don’t get to talk like that, normal.*” (Participant 1, 30 years, facility 1).

In addition to provider continuity and gender, women often expressed a preference for a midwife or nurse rather than physicians for delivery. Some women mentioned that midwives were more sympathetic and caring than doctors.

“*A midwife already knows, that is her job, how to take care of a patient, a doctor has to give different care. In my opinion there should be a midwife, since her job is to take care of women, because midwives are more understanding and more careful than a doctor.*” (Participant 4, 34 years, facility 1).

### Prompt attention

#### Means of transportation and road conditions as determinants of prompt care

In the minority of cases, women living in rural communities far from the facility were able to use their own or their relatives’ means of transportation to reach the facility more quickly and avoid paying for transportation fees.

“[…] *well, I called my uncle, he was at my place with his car, although we had a motorcycle cab at home, but since we were far* [from the hospital], *just in case my uncle said ‘here is the car,’ thank God he took us* [to the hospital]”. (Participant 5, 36 years, facility 1)

However, for most of the women, the lack of transportation was a difficulty in getting to health facilities promptly. Due to the limited availability of transportation, they sometimes had to wait in pain all night until the first trip to the town where the facility was located in the early morning hours. In addition, when women had to pay for public or private transportation to get to the facility, they had to incur very high expenses in relation to their purchasing power. Some women reported having to borrow money to pay for transportation costs. Other participants reported receiving gasoline and food vouchers from the health facility where they gave birth, which was very helpful in keeping them out of debt.

Also, the poor condition of the roads made it difficult to seek for care in a timely and safe manner, especially considering that some women seek care when they are already in pain.

#### Waiting time to initial evaluation upon arrival at the facility

Women’s experiences of waiting to be evaluated upon arrival to the health facility were heterogeneous. Some participants reported that they had been evaluated quickly by health personnel upon arrival —with a reasonable waiting time of about 15 to 30 min according to some participants—, whereas other participants considered that the waiting time had been excessive.

“*Well, they say that it depends on how you go, how long it takes to be attended. Well, I don’t think it should be that long. 15 minutes maximum. Not that they leave us up to an hour, two hours there waiting.”* (Participant 6, 32 years, facility 1).

Participants felt that pregnant women should be given priority at health facilities, especially those in pain. In some of the cases in which waiting time was considered excessive, women stated that patients with less urgent care needs had been attended more promptly, which made them feel upset.

Sometimes, although women were quickly assessed and settled in a bed within the facility, they felt that it took a long time for healthcare staff to perform further revisions.

#### Referral to distant facilities poses a threat to the lives of mothers and babies

Some women reported that the lack of specialists and other resources in the health facilities closest to their homes forced women with complications in pregnancy, labor or childbirth to be transferred to other facilities located between one hour and three hours away, depending on availability, which was perceived as a threat to their lives and those of their babies.

“*In my opinion, the hospital should have a gynecologist or a neonatologist, there should be more doctors specialized in this area, because when something happens the first thing they do is to take their ambulance and go to* [facility 3], *imagine, the road is very bad, the hospital is very far away* […] *when the pregnant woman arrives, the baby has already died or she has already died*.” (Participant 5, 36 years, facility 1).

### Quality of basic amenities

#### Main aspects that determine the comfort of pregnant women in the facility

Some women reported that the facilities where they delivered did not have a conditioned space for waiting, so the users and their companions had to wait outdoors, some of them affected by the cold, heat or rain. The thought of their family members being affected by the inclement weather was a cause of concern during labor for some women.

“[…] *at least there should be a space where family members can stay until it is their turn to come in. For example, that day it rained and my mother was outside in the street waiting for my husband to come out so that she could go in and she was in the rain. And she might have even gotten sick, but it is because there is no room.*” (Participant 7, 34 years, facility 2).

Some women reported that the facilities were too small, including the admission ward, delivery room, postnatal ward, bathrooms, and ambulance, which made them feel uncomfortable. One woman recounted how the small size of the delivery room caused her to hurt herself.

*“It was very small* [the delivery room], *I think this table is narrower, very thin, so only the stretcher could fit, like that table, so tiny, where the legs are placed, which is pure iron that was hurting me.*” (Participant 5, 36 years, facility 1).

Most of the participants considered the premises to be clean. Only one woman noted that the hospital bathrooms were dirty, which made her feel uncomfortable. Apart from the good hygiene of the bathrooms, two women mentioned the importance of these being close to the pregnant women’s beds, to avoid walking long distances in pain and to ensure privacy. Also, some women commented they were annoyed that the bathrooms did not have hot water, making showering uncomfortable.

Some women commented on the importance of having the right temperature at all times in the health premises. Overall, women mentioned that they preferred cooler spaces while waiting for delivery (in the facility or in the maternity waiting home) and during delivery, whereas they preferred warmer spaces when the baby was already born, to prevent him or her from getting cold.

#### Access to food and beverages for users and their relatives

Women were very appreciative of being offered free food and beverages at the health facility, as it saved them both the expense and the difficulty of finding a place with food nearby. Some women, although they refused the food because they had already eaten before arriving at the facility or were not hungry, were grateful for the offer. However, some women who received food from health providers stated that it was not enough to be satisfied or that it was not good.

“[…] *they told me that ‘we brought your food here,’ but it was only enough for two big spoonfuls, ‘that’s what you want me to survive with,’ I told him* […]. *No, maybe, I don’t ask for pure meat, or a big plate, but at least a little more and something tasty. They don’t even salt it, the potato is hard and the pasta is burnt, it’s like they browned it first and then threw it in the water, the pasta was burnt, no flavor.*” (Participant 5, 36 years, facility 1).

Some women expressed concern about the appropriateness of the food provided by the health providers, as it conflicted with their beliefs. One woman suggested that providers receive training on “what is natural”.

*“I imagine that they must have been trained, for example, in the natural way. My experience is that when you give birth, for example, a midwife says ‘you are not going to eat the egg because the egg creates infection flows,’ and there* [at the birthing center] *they gave me an egg because it is different, well, a nurse, from a doctor, from a midwife, from what is natural, and sometimes what is good is the natural, not chemical, and it is different.*” (Participant 7, 34 years, facility 2).

Most of the women who were offered food were not able to choose what they wanted. Some women mentioned that they would have liked chicken broth and/or atole —traditional hot corn gruel from Mexico and some Central American countries— after delivery, as they are believed to be good for breastfeeding.

Participants were also very appreciative when their relatives were offered food and beverages at the facility. One woman, whose relatives have not been offered food, expressed the need for a kitchen or a canteen at the facility where her relatives could cook or buy food while they were waiting for her.

### Access to social support

#### Accompaniment needs during childbirth and its positive aspects

Due to the COVID-19 pandemic, some women were not allowed to bring any companion into the facility during childbirth, which made them feel upset and insecure, especially when they did not know the health personnel. However, in most cases, a companion was allowed in. Women tended to choose their husbands and mothers as companions, as they were the people with whom they felt most comfortable and trustworthy. When the mother or husband was not an option, the mother-in-law, sister, brother, aunt or father were invited as companions. Women appreciated the presence of their mothers because of their experience in giving birth.

“[…] *a mother has experience, she already knows how it feels to be giving birth*.” (Participant 8, 27 years, facility 2).

Some women pointed to male strength as one the reasons they like their husbands to be present.

“[…] [the husband] *helps to support us or hold on because in those moments when I felt pain I would hug the nurse or the midwife to be able to get strength, and a woman is not the same as a man*.” (Participant 9, 22 years, facility 1).

Due to the single-companion restriction, two women were forced to choose between being with their husbands or leaving their other children alone outside the health facility, which was a great concern for them.

“*It was already night and it was raining, and my husband wanted my son to come in and stay with us, they didn’t give him a chance to let my son come in, and the truth is that I was worried inside because where my son was staying, as I didn’t have a relative there… ‘There is no way that my son stays out on the street and with the insecurity there,’ I told him* [the nurse].” (Participant 4, 34 years, facility 1).

Participants considered it important to have companions of their choice during childbirth and reported different positive experiences with them. For instance, the presence of relatives helped women cope with fear and uncertainty. Companions made women feel encouraged, safer and calmer with their presence and support.

“*‘Come on shorty! I love you, you can do it, I know you can, do it for the baby, think of your children. Think of the children left with your mom, they are waiting for you. Come on.’ He hugged me, kissed me, kissed my hands, caressed my hands, rubbed my head.* […] *And yes, his words, his affection gave me a lot of strength, because I was not alone.*” (Participant 5, 36 years, facility 1).

Participants stated that having companions present during childbirth was helpful because they could attend to their needs during the process and help preventing mistreatment by health professionals.

“[…] *the mere fact that I was accompanied by a loved one, well, any pain I might have or any disagreement I might have, I can tell it to a family member and they, they can go out* […]. *The truth is that it is very useful, because it helps us to tell the family member about a doubt we might have, and they can bring us something we need.*” (Participant 4, 34 years, facility 1).

Some women expressed that by accompanying the delivery, the husband could be more involved in the process and empathize with them, which was important to gain their respect and appreciation.

“*For me it was very good, that the husband is present so that he realizes what one suffers, what one goes through to have a baby, not so much so that they love us, but so that they respect us, so that they understand, ‘my wife suffered, I will esteem her, I will respect her, I will pamper her’*”. (Participant 5, 36 years, facility 1)

### Respectful treatment

#### Respectful care for women

Participants felt that pregnant women need special attention from health providers, as they feel pain and sometimes worry or fear.

“*We want a lot of attention at that moment, we are in pain, we want to be checked, to be told how we are doing, how much time we have left, what we need, all that, it is very important at that moment.*” (Participant 10, 34 years, facility 1).

Participants believed that providers should regularly check in on pregnant women and their babies and respond to their questions and concerns. Women, while mentioning that they were aware of the high workload of health providers, often felt that these did not spend enough time with them or were not attentive enough. Participants found it particularly serious that health professionals were more concerned with leisure activities than with caring for pregnant women, especially those who were in pain, hungry or concerned about the health of their baby.

“*And they didn’t come to check me, the doctor was just talking, others were going to paint and they didn’t attend me. That didn’t seem right to me.*” (Participant 8, 27 years, facility 2).

However, most women reported some experiences in which they felt that healthcare providers had been caring, attentive and responsive for them and their newborns.

“*They quickly took me inside and asked me if I wanted a blanket, I was cold and they left me a blanket. They told me that if I needed anything I could tell them what I needed.*” (Participant 2, 29 years, facility 1).

Women also appreciated when health personnel physically helped them if they had to move from one stretcher to another, get up and walk, go to the bathroom or get into the desired position to give birth. In contrast, some women felt annoyed when health providers forced them to walk to speed up the delivery process, despite feeling pain or dizziness.

In addition to being attentive and responsive to women’s needs, women felt it was important that providers performed clinical procedures carefully, taking care not to harm the women or the neonates. Some women reported harm due to some personnel practices, such as vaginal examination, removal of placental debris from the uterus, or intravenous access.

“*They* [the health providers] *would put their hands on me all the time supposedly to clean me and no matter how much I told them not to do it anymore, they kept doing it and it hurt me a lot, they really hurt me a lot, and to date I have just gotten out of bed about a week ago because of the same thing I felt they left me very hurt.*” (Participant 4, 34 years, facility 1).

Women mentioned the importance of health providers being polite when they asked questions and made commentaries. Overall, participants had preference for health professionals that treated them with warmth, which did not limit themselves to provide the service but talk to them. Also, women believed that providers should be supportive and encouraging.

“*The way they treat us should be more pleasant, more polite, just like the doctor, who was spectacular—very affectionate, very kind, very polite—, giving you the confidence that you will be able to make it, that they will indeed support you. If they start saying mean things when you’re in pain, just imagine, they’d kill us.*” (Participant 5, 36 years, facility 1).

Some women reported that they had been reprimanded by health personnel during delivery, which made them feel guilty or affected their self-esteem.

“*Neither I nor my husband liked it* [the treatment from the doctor], *because he was scolding me more than anything else, that how was it possible that I could have a baby weighing 4 kilos and 200 grams, that why didn’t I check myself and why didn’t we check the baby’s weight. It could not be a normal birth, it had to be a cesarean section.* […] *I felt uncomfortable because I was in pain and I didn’t want to hear anything, yes, I felt guilty in part*.” (Participant 10, 34 years, facility 1).

#### Respect and attention for pregnant women’s companions

For participants, respectful treatment by providers was also important for their companions. Some participants reported bad experiences, such as providers “reprimanding” their relatives, while others reported positive experiences. Women appreciated that providers also spoke encouraging words to family members who accompanied them during labor, as well as cared for them by offering water, food, or rest.

“*They were also giving him* [her husband] *words of encouragement, telling him if he was tired to go rest for a while that they were going to stay and take care of me. My mom the same, that if she was hungry, if she wanted water, if she wanted something, she could ask for it.*” (Participant 8, 27 years, facility 2).

### Privacy

#### Exposure of women’s bodies to the gaze of strangers

Many women reported feeling their bodies exposed to the gaze of strangers while waiting to give birth, which made them very uncomfortable. Some women were left in the corridor on a stretcher in full view of other health providers, patients and their companions. Other women expressed they felt uncomfortable waiting for delivery in a general ward with non-pregnant patients —including men and women— and their relatives. Women felt especially uncomfortable when undergoing vaginal examinations exposed to the gaze of others.

“*In the hospital it had to be… at least for me when they did the examination, there were sometimes two doctors, and it’s not like they were going to put you on the stretcher and close the curtain, no, sometimes they closed it and sometimes they didn’t, you were left in the open air. And they are passing by and passing by, and they are watching you like this, with the doctors checking you.*” (Participant 1, 30 years, facility 1).

For some participants, being observed by more providers than perceived as necessary represented a violation of their privacy.

“*What I didn’t like, was that where they had me lying down there were… Some guys, some nurses learning, they were watching. There were about three people besides the doctor and the other nurse. There were three others in there. They were watching how the process was going to go. I don’t know. But I felt uncomfortable in there, at that instant.*” (Participant 11, 20 years, facility 3).

Several women also reported feeling uncomfortable in the postnatal ward when other women’s relatives were nearby, sometimes without a curtain.

In addition, some participants stated that it was important that health providers gave them gowns that adequately covered them, as they felt embarrassed when they had to stand up and their bodies were exposed in front of strangers due to the opening of the gown.

### Involvement in decisions

#### Informed consent before health providers perform clinical interventions

Several participants noted as a key aspect of respect for women’s will that, before performing an intervention deemed necessary for clinical reasons, health personnel informed women about it and asked for their consent to proceed. Some women narrated positive experiences in this regard.

“*Everything they did they explained to me what for, why and how and all that, although not about the baby. They did the touching, they told me what it was for, in fact, they asked me and my husband for permission because that’s uncomfortable. I think it’s not allowed anymore. And I said yes, I wanted to know how far along I was.*” (Participant 5, 36 years, facility 1).

In situations where this did not occur, participants considered the practices rude, careless and an invasion of their privacy. Participants complained that providers did not ask for consent before performing vaginal examinations, episiotomies, removal of placental debris or cannulations.

“*The doctor told me to spread my legs and she didn’t even say ‘I’m going to do this to you.’ […] She didn’t ask ‘ma’am, let me have your legs or I’m going to do this to you.’ She abruptly gave me the tact.*” (Participant 12, 29 years, facility 1).

#### Aspects of delivery care important to decide other than clinical interventions

In addition to deciding what clinical interventions they wanted to have performed, participants expressed a number of other important choices to make during delivery. The most common aspects reported by the women were deciding whether to have a companion, what and when they wanted to eat and drink, what position to give birth in, when to walk, when to shower, when to go to the bathroom, and what to wear. For all these aspects there was more than one woman who was denied to decide according to her preferences.

In terms of choice of delivery position, permissiveness varied from facility to facility. For instance, most women who gave birth at the birthing center reported that they were offered a choice from a list of birthing position options. Despite having a choice, many women decided to have their babies lying on a stretcher, as this was the way they had had their previous deliveries in most cases, especially those who had delivered in hospitals, where a choice of birthing position is rarely offered. However, regardless of the position chosen, most women appreciated that providers gave them the possibility to choose the position of their preference. One woman who was not allowed to give birth in the position she wanted relayed her experience.

“*Well, I wanted to have my baby lying down, but they wouldn’t let me. They said no because they told me that she would fall and hurt herself or something like that, because you can’t get any strength on the stretcher like that.*” (Participant 13, 19 years, facility 5).

### Communication

#### Provision of information by healthcare providers

Participants appreciated when health providers provided information —both answering to women’s questions or without its request— such as what to expect during the different stages of delivery, how to breathe, the reasons for clinical procedures, causes of physiological reactions such as excessive bleeding or pain, and how to stimulate breastfeeding. Some primipara women expressed that it was key that providers explained everything to them because it was their first time giving birth and they had many questions. Having providers offer understandable information made participants feel good, more confident, trusting, and calmer. Some women reported satisfactory explanations from health providers.

“*Well, every time they checked me they always explained to me how much I had, what I had, what I could do, and so they always told me that if I had any doubts I should tell them* […]. *Every time they checked me they always asked me questions, I felt good because I didn’t have any doubt.*” (Participant 8, 27 years, facility 2).

In some cases, women also reported experiences of poor communication, sometimes between providers and family members waiting outside the facility, leading to confusing situations.

#### Information requested by health providers at inappropriate times

Some participants considered inappropriate the request of information by providers after admission to the health facility. Three women expressed discomfort when demanded for information while in pain.

“*And what… what I didn’t like was that you are still in pain, they still ask you what your name is or where you come from, because it is a strong pain and you have to put up with a lot and they are still asking; you don’t even want to answer.*” (Participant 9, 22 years, facility 1).

#### Communication between providers influences user experience

Communication between health providers in front of the women receiving delivery care also influenced their experience. One woman recounted an argument between providers in front of her that made her feel uncomfortable.

“*They were going to deliver a shift change and the charge nurses said, ‘no, it’s wrong here. Call the doctor. And why didn’t you tell her how many children you have?’ ‘Yes, I did,’ I said. Then, the little doctor* [“doctorcito” in original transcription] *came and corrected it. However, for them* [the nurses] *everything was wrong. They started to argue that they* [the doctors] *always don’t do things right.*” (Participant 5, 36 years, facility 1).

## Discussion

This study presents new insights into how non-clinical aspects of care shape the experience of facility-based delivery in the state of Chiapas, focusing on the eight health systems responsiveness domains described by the WHO. Through the findings of our study, we shed light on the barriers women face in receiving prompt care, aspects of health facilities that impact women’s comfort, the relevance of being provided with adequate food and drink during institutional delivery, how accompaniment contributes positively to the birthing experience, the aspects of childbirth that women find important to decide on, and how providers’ interpersonal behaviors affect the birthing experience.

One of the main concerns of the participants was the multiple barriers that prevented them from receiving prompt delivery care. The lack of facilities in the region, the unavailability of transportation, its cost, and poor road conditions made it difficult for pregnant women to access health facilities in a timely manner, as previously reported in Chiapas [28, 35]. Once at the chosen facility, due to lack of human resources and equipment, women received care with delays or, when complications arose, had to be referred to distant institutions, again facing all the transportation barriers. In line with our study, waiting time for care and speed of referrals to other health facilities have been identified as determinants of the birthing experience and user satisfaction in other studies conducted in low-resource settings [36], where delays in delivery care due to access barriers and lack of resources are common [37, 38].

Study participants emphasized the importance of some aspects of the facilities for their comfort, the most common being hygiene (satisfactory in most cases), having hot water for showering, availability of a waiting area, adequate room space, and adequate temperature. The facility physical environment for maternal healthcare service users has previously been identified as a key determinant of user satisfaction in other low-resource settings, specifically seating and waiting areas, adequate room space, electricity, water supply, bed comfort, cleanliness, ambient lightning, and ambient sound [36, 39]. Although resource constraint may make it difficult to address all of these issues, knowledge of user preferences can be helpful in prioritizing which aspects to improve first.

Women also emphasized the importance of being provided food and drink during their stay at the facility to have a satisfactory birth experience. Some participants reported that they had not received good and/or sufficient food during their stay at the health facility, which negatively affected their childbirth experience, as mentioned in another qualitative study [40]. In addition, some women mentioned the importance of being provided with specific food choices in line with their beliefs, as according to the popular culture of the study setting there are some foods and beverages that are recommended for better mother and neonate outcomes, while other should be avoided [41]. Cultural sensitivity in the food provided in healthcare settings has been underscored before as an important element in patient care [42].

Another aspect relevant to the birth experience of the participants was the possibility of having a companion with them. Women highlighted some of the beneficial aspects of accompaniment, such as making them feel encouraged and calmer, being supportive in communicating with providers, preventing mistreatment, and attending to their needs. A study focused in women’s perceptions of birth accompaniment reported similar perceived benefits, adding an interesting theme that did not emerge in our study: intimate partner accompaniment as an expression of shared responsibility of pregnancy and potential parenthood [43].

Women considered it important that their preferences be taken into account throughout the delivery, respecting their right to make autonomous decisions. Some of the most important aspects to decide according to the participants were the professional who would attend their childbirth (preferably female nurses or midwifes they knew), giving consent for clinical procedures, how and when to move, the birthing position, and what to wear. Most participants preferred to be attended by a female professional, which is consistent with preferences expressed in other studies worldwide [36], sharing reasons such as greater communication, a greater sense of privacy, and a greater understanding of the women’s needs. Our findings resonate with a study conducted with indigenous women in Chiapas, in which the selection of the provider (female traditional midwifes from their communities), the birthing position, and what to wear where key aspects of the facility-based childbirth experience, allowing users to give birth in congruence with their traditional customs and practices [12]. As in our study, the lack of respect for women’s autonomy during institutional childbirth has been widely reported in vulnerable populations in Chiapas [12, 29, 30, 44], including such serious cases as the application of fertility regulation methods without the woman’s consent.

Most of the participants in our study agreed on the importance of health providers being attentive, caring, polite, communicative, respectful of their privacy, and supportive, both to themselves and to those accompanying them. Interpersonal behavior has been identified as a major determinant of women’s satisfaction with childbirth care in other studies, which cite respect, courtesy, being caring, politeness, and active listening as some of the key aspects of provider care [36]. However, cases of mistreatment and miscommunication toward women and their companions have been commonly reported in our study and other studies in the region [9, 44] and Mexico [45, 46], negatively affecting women’s experiences. In our study, disrespectful behaviors included reprimands, harm, omission, and disrespect for women’s privacy. These behaviors may deter women from using maternal healthcare services in the future [47, 48].

By recalling participants’ experiences and perceptions of care during childbirth, it was possible to identify non-clinical aspects of institutional delivery care that are important to users and need to be improved in public health facilities. Improving these factors would lead to greater responsiveness of health services, which can ultimately enhance health service utilization and user health outcomes [20].

Our study is not without limitations. The association by participants of the data collector with CES –the NGO providing care at the birthing center where participants were recruited– could have resulted in social desirability bias. To reduce this source of bias, patients were assured of confidentiality and voluntary participation. In addition, the questions were designed to elicit sincere answers. Another limitation is the fact that participants gave birth during the COVID-19 pandemic, which may limit the generalizability of the findings to other periods, as in the first semester of 2022 healthcare facilities were still affected by resource shortages related to the pandemic response [49] and healthcare providers had higher than usual levels of stress [50].

## Conclusions

Our study sheds light on the experiences, perceptions, and preferences of users of childbirth care services in public health institutions in Chiapas. We have identified non-clinical aspects of childbirth care that are relevant to users and that are not being satisfactorily addressed by healthcare providers and institutions in the state, including prompt access to care, participation in decisions, respectful treatment and communication, privacy, and comfort of amenities. This evidence constitutes a necessary first step towards the design of strategies to improve the responsiveness of the Chiapas health system in childbirth care and to guarantee women’s rights.

### Electronic supplementary material

Below is the link to the electronic supplementary material.

Supplementary Material 1

Supplementary Material 2

## Data Availability

The data presented in this study are available on request from the corresponding author. The data are not publicly available due to privacy and confidentiality.

## Abbreviations

CES: Compañeros En Salud
INSP: National Institute of Public Health of Mexico
MOH: Ministry of Health
NGO: Non–Governmental Organization
WHO: World Health Organization
