# Supplementary material for: Exploring how non-clinical factors in childbirth care shape users’ experiences in public health facilities in rural Chiapas, Mexico: a qualitative study using the WHO health systems responsiveness framework
Source: BMC Pregnancy Childbirth. 2024 Feb 29;24:173. doi: 10.1186/s12884-024-06357-7 (PMC10905866; doi:10.1186/s12884-024-06357-7)
Supplement: Supplementary file 1 — Supplementary Material 1 [file 12884_2024_6357_MOESM1_ESM.pdf]

Consolidated criteria for reporting qualitative studies (COREQ): 32-item checklist

No	Item	Guide questions/description	Page where the information is included
**Domain 1: Research team and reflexivity**			
Personal Characteristics			
1.	Interviewer/facilitator	Which author/s conducted the interview or focus group?	7
2.	Credentials	What were the researcher's credentials? *E.g. PhD, MD*	7
3.	Occupation	What was their occupation at the time of the study?	7
4.	Gender	Was the researcher male or female?	7
5.	Experience and training	What experience or training did the researcher have?	7
Relationship with participants			
6.	Relationship established	Was a relationship established prior to study commencement?	7
7.	Participant knowledge of the interviewer	What did the participants know about the researcher? e.*g.*	7

No	Item	Guide questions/description	Page where the information is included
		personal goals, reasons for doing the research	
8.	Interviewer characteristics	What characteristics were reported about the interviewer/facilitator? e.g. *Bias, assumptions, reasons and interests in the research topic*	29
**Domain 2: study design**			
Theoretical framework			
9.	Methodological orientation and Theory	What methodological orientation was stated to underpin the study? *e.g. grounded theory, discourse analysis, ethnography, phenomenology, content analysis*	5-6
Participant selection			
10.	Sampling	How were participants selected? *e.g. purposive, convenience, consecutive, snowball*	6
11.	Method of approach	How were participants approached? e*.g. face-to-face, telephone, mail, email*	6

No	Item	Guide questions/description	Page where the information is included
12.	Sample size	How many participants were in the study?	6
13.	Non-participation	How many people refused to participate or dropped out? Reasons?	6
Setting			
14.	Setting of data collection	Where was the data collected? e.*g. home, clinic, workplace*	6
15.	Presence of non-participants	Was anyone else present besides the participants and researchers?	6
16.	Description of sample	What are the important characteristics of the sample? *e.g. demographic data, date*	8-9
Data collection			
17.	Interview guide	Were questions, prompts, guides provided by the authors? Was it pilot tested?	7
18.	Repeat interviews	Were repeat interviews carried out? If yes, how many?	7
19.	Audio/visual recording	Did the research use audio or visual recording to collect the data?	7

No	Item	Guide questions/description	Page where the information is included
20.	Field notes	Were field notes made during and/or after the interview or focus group?	7
21.	Duration	What was the duration of the interviews or focus group?	7
22.	Data saturation	Was data saturation discussed?	6
23.	Transcripts returned	Were transcripts returned to participants for comment and/or correction?	6-7
**Domain 3: analysis and findings**z			
Data analysis			
24.	Number of data coders	How many data coders coded the data?	7-8
25.	Description of the coding tree	Did authors provide a description of the coding tree?	7-8
26.	Derivation of themes	Were themes identified in advance or derived from the data?	7-8
27.	Software	What software, if applicable, was used to manage the data?	7-8

No	Item	Guide questions/description	Page where the information is included
28.	Participant checking	Did participants provide feedback on the findings?	7-8
Reporting			
29.	Quotations presented	Were participant quotations presented to illustrate the themes / findings? Was each quotation identified? e.*g. participant number*	8-25
30.	Data and findings consistent	Was there consistency between the data presented and the findings?	8-25
31.	Clarity of major themes	Were major themes clearly presented in the findings?	8-25
32.	Clarity of minor themes	Is there a description of diverse cases or discussion of minor themes?	8-25
