# Supplementary material for: Exploring how non-clinical factors in childbirth care shape users’ experiences in public health facilities in rural Chiapas, Mexico: a qualitative study using the WHO health systems responsiveness framework
Source: BMC Pregnancy Childbirth. 2024 Feb 29;24:173. doi: 10.1186/s12884-024-06357-7 (PMC10905866; doi:10.1186/s12884-024-06357-7)
Supplement: Supplementary file 2 — Supplementary Material 2 [file 12884_2024_6357_MOESM2_ESM.pdf]

ORIGINAL INTERVIEW GUIDE IN SPANISH

1 EXPERIENCIA DE SU ÚLTIMO PARTO

1.1 En su último parto, ¿cómo fue el proceso desde que empezó con el trabajo de parto, hasta que tomó la decisión de acudir al centro de salud, hasta su llegada al centro de salud? ¿Qué tiempo pasó entre el inicio del trabajo de parto y la decisión de acudir al centro de salud? ¿Y entre que decidió ir al centro de salud y que llegó al centro de salud? ¿Qué barreras se encontró para poder acceder al centro de salud? *Ejemplo: Barreras económicas, de transporte, oposición de algún miembro de la familia…* ¿Cómo le hizo sentir este proceso? ¿Por qué?

1.2 Una vez llegó al centro de salud, ¿cómo fue la experiencia de su último parto? Explíqueme todo el proceso, desde que estaba esperando a que la atendieran hasta que se marchó con su bebé del centro de salud. ¿Cómo se sintió de forma general? ¿Por qué?
Ejemplo: insegura/cuidada

1.3 ¿Cómo fue la comunicación con el personal de salud? ¿Tuvo dificultades para comprender lo que decían? ¿Cómo actuaban cuando usted les preguntaba algo? ¿Cómo la hizo sentir esta comunicación con el personal de salud? ¿Por qué?

1.4 ¿Cómo la trató el personal de salud a usted y a su familia durante la atención de su último parto? ¿Considera que recibió alguna forma de maltrato durante la atención? ¿Cuál? ¿Cómo la hizo sentir este trato? ¿Por qué?

1.5 ¿Durante el parto, usted pudo decidir quién la atendía? *Ejemplo: tipo de personal (incluyendo apoyo de partera tradicional), edad y género.* ¿Cómo la hizo sentir poder elegir/no poder elegir quién atendía su parto? ¿Por qué?
¿Pudo elegir la forma en la que tenía su parto? ¿Cuáles fueron las cosas que el personal de salud le dejó elegir? *Qué posición de parto, qué acompañantes, qué vestimenta, qué comida, cuándo pararse a caminar, qué ejercicios relajantes hacer...* ¿Cómo la hizo sentir poder elegir/no poder elegir cómo la atendían? ¿Por qué?

1.6 ¿Cómo fueron los tiempos de espera para que la atendieran durante las diferentes etapas del parto? ¿Qué duración tuvieron? ¿Cómo la hicieron sentir? ¿Por qué?

1.7 ¿Tuvo acompañamiento de sus familiares u otras personas allegadas durante el parto? Si lo hubo, ¿cómo fue? El hecho de que tuviera/no tuviera acompañamiento, ¿cómo la hizo sentir? ¿Por qué?

1.8 ¿Cómo se respetó su intimidad durante el parto? ¿Cómo la hizo sentir? ¿Por qué?

1.9 ¿Cómo eran las instalaciones donde dio a luz? ¿Y otras instalaciones del centro de salud de las que hiciera uso justo antes o después de dar a luz? *Como albergue, sala de recuperación…* ¿Cómo eran la iluminación, la ventilación, la distancia con otras personas, la comodidad, los sanitarios, la comida, la higiene general…? ¿Cómo la hicieron sentir? ¿Por qué?

2 PREFERENCIAS

2.1 Para usted, ¿cómo sería una atención de parto de buena calidad en un centro de salud?

¿Y de mala calidad?

2.2 De acuerdo a la experiencia de su último parto, ¿podría darnos ejemplos donde recibió una atención de buena calidad? ¿Y de mala calidad?

2.2 Para recibir atención de parto de buena calidad en un centro de salud, ¿cómo debería ser la comunicación con el personal de salud?

2.3 Para recibir atención de parto de buena calidad en un centro de salud, ¿cómo debería ser el trato del personal de salud hacia usted y sus familiares?

2.4 Para recibir atención de parto de buena calidad en un centro de salud, ¿qué cosas debería poder elegir según su preferencia? *Ejemplo: quién atiende el parto, posición de parto, acompañantes, vestimenta, comida, cuando pararse a caminar, ejercicios relajantes...*

2.5 Para recibir atención de parto de buena calidad en un centro de salud, ¿cómo deberían ser los tiempos de espera para ser atendida?

2.6 Para recibir atención de parto de buena calidad en un centro de salud, ¿cómo debería ser el acompañamiento de sus familiares/allegados?

2.7 Para recibir atención de parto de buena calidad en un centro de salud, ¿cómo deberían ser el lugar y el trato para respetar su intimidad?

2.8 Para recibir atención de parto de buena calidad en un centro de salud, ¿cómo deberían ser las instalaciones? *Ejemplo: iluminación, higiene, sanitarios, comodidad… Incluir el albergue y/o sala de recuperación si procede.* ¿Y la comida?

3 ÁREAS DE MEJORA

3.1 Si tuviera oportunidad de cambiar algo en su último parto, ¿qué cambiaría?
Ejemplo: personal de salud, comida, instalación, acompañamiento, ruido, privacidad, etc.

3.2 Si tuvo más partos naturales/vaginales antes, ¿dónde fueron sus otros partos? ¿Qué le pareció mejor que en su último parto? ¿Qué le pareció peor que en su último parto? ¿Por qué?

4 PREFERENCIA DE LUGAR DE PARTO

4.1 En su último embarazo, ¿dónde quería tener su parto inicialmente? ¿Por qué?
Finalmente lo tuvo en *indicar nombre de la institución donde tuvo el parto,* ¿por qué? ¿Cómo la hizo sentir poder/no poder tener su parto donde le habría gustado?

4.2 ¿Si volviera a tener un bebé dónde le gustaría atender su parto? ¿Por qué?
Ejemplo: en casa o en una determinada institución de salud.

4.3 ¿Si una mujer fuera a tener su parto y le preguntara cuál es el mejor lugar para hacerlo, qué respondería? ¿Por qué?

INTERVIEW GUIDE TRANLATED TO ENGLISH

1 EXPERIENCE OF YOUR LAST CHILDBIRTH

1.1 In your last childbirth, what was the process like from the time you went into labor, until you made the decision to go to the health facility, to your arrival at the health facility? How long did it take between the onset of labor and the decision to go to the health facility? And between the time you decided to go to the health facility and the time you arrived at the health facility? What barriers did you encounter in accessing the health facility? *Example: Economic barriers, transportation barriers, opposition from a family member...* How did this process make you feel? Why?

1.2 Once you arrived at the health facility, how was the experience of your last delivery? Explain the whole process, from when you were waiting to be seen until you left the health facility with your baby. How did you feel in general? Why? *Example: Unsafe/cared for*

1.3 How was the communication with the health personnel? Did you have difficulty understanding what they were saying? How did they act when you asked them something? How did this communication with the health staff make you feel? Why?

1.4 How did health care staff treat you and your family during your last delivery? Do you think you were abused in any way during care? Which one? How did this treatment make you feel? Why?

1.5 During delivery, were you able to decide who cared for you? *Example: type of staff (including traditional birth attendant support), age, and gender.* How did it feel to be able to choose/not be able to choose who gave you care? Why? Were you able to choose the way you delivered? What were the things the health staff let you choose? *What birthing position, what companions, what clothes, what food, when to walk, what relaxing exercises to do...* How did it make you feel to be able to choose/not be able to choose how you were cared for? Why?

1.6 What were the waiting times like for you to be seen during the different stages of labor? How long did they last? How did they make you feel? Why?

1.7 Were you accompanied by family members or other close people during delivery? If so, what was it like? The fact that you had/didn't have accompaniment, how did it make you feel? Why?

1.8 How was your privacy respected during delivery? How did it make you feel? Why?

1.9 What were the facilities like where you gave birth? What about other facilities that you used just before or after giving birth? *As a shelter, recovery room...* What were the lighting, ventilation, distance from other people, comfort, toilets, food, general hygiene...? How did they make you feel? Why?

2 PREFERENCES

2.1 What would good quality birth care look like for you in a health facility?

And poor quality?

2.2 Based on the experience of your last birth, could you give us examples where you received good quality care? And poor quality?

2.2 To receive good quality childbirth care in a health facility, what should communication with health personnel look like?

2.3 To receive good quality childbirth care in a health facility, how should health personnel treat you and your family members?

2.4 To receive good quality birth care at a health facility, what things should you be able to choose based on your preference? *Example: who attends the birth, birthing position, companions, clothing, food, when to walk, relaxing exercises...*

2.5 To receive good quality childbirth care in a health facility, what should wait times be like?

2.6 To receive good quality birth care in a health facility, what should the accompaniment of your relatives/friends be like?

2.7 To receive good quality childbirth care in a health facility, how should the place and treatment be to respect your privacy??

2.8 To receive good quality birth care in a health facility, what should the facilities look like? *Example: lighting, hygiene, sanitaryware, comfort... Include the shelter and/or recovery room if applicable.* And the food?

3 AREAS FOR IMPROVEMENT

3.1 If you had the opportunity to change something in your last birth, what would you change? *Example: health personnel, food, accompaniment, noise, privacy, etc.*

3.2 If you had more natural/vaginal births before, where were your other births? What did you think was better than your last birth? What did you think was worse than your last birth? Why?

4 BIRTHING SITE PREFERENCE

4.1 In your last pregnancy, where did you want to have your birth initially? Why?
Finally you gave birth in *indicate the name of the institution where she gave birth*, why?
How did it make you feel to be able/unable to have your birth where you would have liked?

4.2 If you were to have a baby again, where would you like to deliver? Why?
Example: at home or in a certain health institution.

4.3 If a woman were to give birth and asked you where is the best place to do it, what would you answer? Why?
